# Evaluation of secondary metabolites, antioxidant activity, and color parameters of Nepali wines

**DOI:** 10.1002/fsn3.794

**Published:** 2018-10-10

**Authors:** Ankit Pandeya, Sagar Rayamajhi, Pravin Pokhrel, Basant Giri

**Affiliations:** ^1^ Center for Analytical Sciences Kathmandu Institute of Applied Sciences Kathmandu Nepal; ^2^ Present address: Department of Chemistry University of Kentucky Lexington Kentucky; ^3^ Present address: Department of Chemistry Kansas State University Manhattan Kansas

**Keywords:** anthocyanins, antioxidants, Nepal, red wine, tannins, wine color

## Abstract

We evaluated the quality of wines produced in Nepal in terms of phenolic, flavonoid, anthocyanin and tannin content, antioxidant capacity, and color parameters using spectrophotometric methods. The total phenolic content, total flavonoid content, and total antioxidant activities in Nepali wines ranged from 85.5 to 960.0 (mean = 360.5 ± 268.7) mg/L GAE, 40.9–551.3 (mean = 188.9 ± 161.5) mg/L QE, and 66.6–905.0 (mean = 332.8 ± 296.5) mg/L AAE, respectively. These parameters were significantly higher in red wines compared to white wines. The phenolic and flavonoid content showed strong correlation with each other as well as with antioxidant activities. Additional parameters measured included various color parameters and carbohydrates. The wine color showed strong correlation with phenol, flavonoid, and antioxidant activity, whereas this correlation was not significant with anthocyanin content. Multivariate analysis was carried out to better describe and discriminate the wine samples. Finally, we compared Nepali wines with wines from other countries.

## 1 INTRODUCTION

Wine is considered as one of the most consumed and prehistoric alcoholic beverages. This widely celebrated alcoholic drink across the globe is primarily made from grapes but several other fruits and berries, including apple, pear, peach, blackberry, raspberry, strawberry, cherry, blueberry, plum, banana, and mango, are also being used. In many cases, additional supplements such as sucrose, honey, medicinal herbs, and local fruits are also practiced to make wines having higher medicinal value with characteristic flavor (Davidović et al., 2013; Jagtap & Bapat, 2015).

Compared to other alcoholic beverages, wines are particularly important than other alcoholic beverages because of a variety of secondary metabolites that provide multiple health benefits. Red wines are found to have higher in vitro free radical scavenging activity making them an important source of natural antioxidants (De Beer, Joubert, Gelderblom, & Manley, 2003) than beverages such as beer, tea, and fruit juices. Besides, several health benefits of wine have been reported against conditions such as cardiovascular diseases, atherosclerosis, cancer, neurological degeneration, gastrointestinal infections, diabetes, hypertension, and pulmonary disorders (Guilford & Pezzuto, 2011). Similarly, red wines have been reported to ameliorate the effects of oxidative stress by increasing plasma antioxidant capacity, suppressing reactive oxygen species generation, increasing serum oxygen radical absorbance capacity, and decreasing oxidative DNA damage (Guilford & Pezzuto, 2011). Identified as one of the important constituents of wine, resveratrol is reported to exhibit cardioprotective and chemopreventive effects by altering lipid metabolism, inhibiting low‐density lipoprotein oxidation, and inhibiting platelets aggregation in animal (Guerrero, Garcia‐Parrilla, Puertas, & Cantos‐Villar, 2009).

Medicinal value of wine is primarily derived from secondary metabolites. Among various secondary metabolites, phenolic compounds are the principal bioactive components of wines (Shahidi & Ambigaipalan, 2015). There are over 200 phenolic compounds identified in wines. In general, phenolic compounds have one or more hydroxyl groups present in the aromatic rings. These hydroxyl groups play a major role in attributing antioxidant properties by scavenging reactive species. Phenolic compounds present in wines can be broadly classified as flavonoids (e.g., flavanols, flavonols, anthocyanins, and tannins) and nonflavonoids (e.g., hydroxybenzoic acids, hydroxycinnamic acid derivatives, and stillbenes) based upon their carbon skeleton (Cosme, Gonçalves, Inês, Jordão, & Vilela, 2016; De Beer et al., 2003). Composition and amount of phenolic compounds in wines vary greatly with the raw materials used, climatic condition, and soil environment where the raw materials are grown, wine‐making procedure, storage, and aging of wine. Furthermore, the quantity of these bioactive compounds depends on several parameters such as temperature, fermentation vessel, duration of maceration, yeast strain used, SO_2_, pH, and pectolytic enzymes during vinification (Stratil, Kuban, & Fojtova, 2008).

Quality of wines is primarily determined based on chemical composition, color, and aroma. Color and aroma give the first and foremost characteristic of wines. Color is also analyzed to derive information on defects, degree of phenolic composition, quality of wine preservation, and storage duration (Sáenz‐Navajas, Echavarri, Ferreira, & Fernández‐Zurbano, 2011). In addition, color accounts for overall acceptability by consumers. Normally, anthocyanins are associated with wine color and other phenolic compounds such as catechins, epicatechins, and tannins are related to bitterness and astringency (Glories, 2000; Mazza & Francis, 1995; Ribéreau‐Gayon & Glories, 1986; Salinas, Schaeffer, Maréchal‐Drouard, & Duchêne, 2005). Screening of various parameters in wines and other food products is on rise in recent years because of their possible role in better health, nutritional value, and disease prevention. There are several reports on the quality of commercially produced wines from various countries such as Italy (Cassino et al., 2016; Garaguso & Nardini, 2015), China (Jiang & Zhang, 2012; Li, Wang, Li, Li, & Wang, 2009), Australia (Yoo, Prenzler, Saliba, & Ryan, 2011), Brazil (Gris et al., 2013), Czech Republic (Stratil et al., 2008), Portugal (Stratil et al., 2008), Romania (Hosu, Cristea, & Cimpoiu, 2014), and Spain (Gómez‐Plaza, Olmos, & Bautista‐Ortín, 2016). Screening of the parameters such as total phenols on commercial wine also helps to assess the quality wines and allocate them into different grades and price. However, there are no scientific studies on commercial Nepali wines in terms of their quality parameters.

Modern wine production is relatively new in Nepal. Past decade has seen a significant rise in the consumption of wine in Nepal with about 15 million liters in 2016 (Rijal, 2016). Domestic wines occupy ~35% of total wine market in Nepal (Basnet, 2016). Nepali wines are claimed to have characteristic flavor contributed by local raw materials and high altitude vinification. In this work, we evaluated the total phenols, flavonoids, tannins, anthocyanins, carbohydrates, and antioxidant capacity of 19 different commercial Nepali wine samples. We also evaluated a number of color parameters to understand their relationship with secondary metabolites. Finally, we present a comparison of selected parameters from our work to those from studies carried out in commercial wines produced in other countries.

## 2 MATERIALS AND METHODS

### 2.1 Chemicals and reagents

Gallic acid, ascorbic acid, quercetin, and 1,1‐diphenyl‐2‐picrylhydrazyl (DPPH) were purchased from Hi‐Media Laboratories, India. Folin–Ciocalteu reagent, sodium carbonate, methanol, ethanol, sodium nitrite, sodium bisulfite, anthrone, sulfuric acid, D‐glucose, and hydrochloric acid were purchased from Thermo Fisher Scientific, India. Similarly, aluminum chloride was purchased from SD Fine‐Chem Limited, India, and acetaldehyde was purchased from Nike Chemicals, India.

### 2.2 Wine samples

We purchased 19 commercial wines (12 red wines and seven white wines) from local liquor stores in Kathmandu, the capital city of Nepal. The wine samples represented ten different manufactures in Nepal. According to the labels in the wine bottles, average maximum retail price per bottle wine was 4.2 USD (max = 5.6, min = 3.3) in current currency exchange rate ($1 = NRs101.9). The age of wine samples ranged from freshly prepared to <2 years at the time of analysis. Reported alcohol content ranged from 9% to 12%. Each sample was given a code before analysis. The measured pH of the wine samples was found to be 3.29 ± 0.14 (range: 2.93–3.54). As reported in the label, following fruits were commonly used to make the wines: grapes, palm, pear, fruit roots, herbs, honey, tea leaves, spices, flowers, chutro (barberry), aiselu (raspberry), orange, pineapple, apple, apricots, mountain berries, banana, plum, lime, and lychee fruit. Description of these wine samples is given in Table 1. The wine bottles were stored in dark and analyzed immediately after opening or stored at 4°C in the laboratory until analyzed.

**Table 1:** Description of wine samples. Information given in this table, except pH values, was obtained from label on the bottle of wines

Sample ID	Brand name	Mfg. date	MRP (USD)	Type	Alcohol (%)	pH	Raw materials used
W01R	Dandaghare	November, 2015	4.2	Red	11.6	3.22	Fruits, roots, herbs, and honey from Himalayan, Hilly, and Tarai regions
W02W	Dandaghare	November, 2015	4.2	White	11.9	3.27	Fruits, roots, herbs, and honey from Himalayan, Hilly, and Tarai regions
W03R	Hinwa	September, 2016	4.0	Red	11.5	3.35	Chutro (barberry), aiselu (Himalayan Raspberry)
W04W	Hinwa	September, 2016	4.0	White	11.5	3.54	Aiselu (Himalayan Raspberry)
W05R	King's Hill	October, 2016	4.6	Red	10.0	3.54	Grapes
W06W	King's Hill	October, 2016	4.6	White	10.0	3.28	Apple
W07R	Nesy	March, 2016	3.8	Red	11.5	3.22	Blend of grape and apple
W08W	Nesy	February, 2016	3.8	White	11.5	3.17	Grape and apple
W09R	Royal big master	2015	4.1	Red	9–10	3.32	Grape
W10R	Royal big master	December, 2015	4.8	Red	9–10	3.26	Grape
W11W	Royal big master	April, 2015	4.1	White	11–12	3.35	Apple, pineapple, grapes
W12R	Royal big master	March, 2016	4.8	Red	11–12	3.39	Grapes
W13R	Oldcity's empire	July, 2015	3.4	Red	11.5	3.29	Banana and grapes with added plum and lime
W14R	JB Wine	2015	4.0	Red	11.5	2.93	Orange, pineapple, grapes, apple, apricots, and mountain berries
W15R	Divine ultrablack	July, 2015	4.1	Red	11.5	3.33	Tea leaves, fruits, spices, and flowers
W16R	Chesung	2016	3.8	Red	11.5	3.13	Natural palm, pear, and grapes
W17R	Oldcity's valentine	December, 2016	5.8	Red	11.5	3.26	Tempranillo grape
W18W	Royal big master	April, 2016	4.8	White	11–12	3.43	Apple
W19W	Divine	October, 2014	5.5	White	11.5	3.23	Lychee fruit

Mfg.: manufactured; MRP: maximum retail price.

### 2.3 Assay methods

All spectrophotometric measurements were performed in Epoch Microplate Spectrophotometer (BioTech Instruments Inc., Winooski, VT, USA).

#### 2.3.1 Total phenol content

The total phenolic content (TPC) was determined by Folin–Ciocalteu (FC) method (Moreno‐Arribas & Polo, 2009). In brief, 200 μl of FC reagent (10% v/v) was added to 100 μl of wine sample previously diluted as required (10‐fold dilution for red wines and fivefold for white wines based on preliminary experiments). After allowing the reaction mixture to react for 5 min, 800 μl sodium carbonate (7.5% w/v) was added. The whole mixture was then incubated at room temperature in dark for 2 hr. The mixture was then centrifuged at 2,520 *g* for 4 min and 200 μl of the supernatant was transferred into the microplate well to measure the absorbance at 725 nm against a blank containing methanol in place of wine sample. Phenolic content of the wine sample was expressed as mg/L gallic acid equivalent (GAE) using a calibration curve in the concentration range 7.5–125 mg/L.

#### 2.3.2 Total flavonoid content

The total flavonoid content (TFC) was determined by AlCl_3_ method (Moreno‐Arribas & Polo, 2009). In brief, 75 μl of wine was mixed with 75 μl of NaNO_2_ (6 g/L) in a microplate well. After 5 min, 75 μl of AlCl_3_ (22 g/L) was added to the mixture. The reaction was left for completion for 5 min, and absorbance was measured at 425 nm against an ethanolic blank (80% ethanol). Flavonoid content was quantified as mg quercetin equivalent/L of wine using calibration curve obtained in the concentration range of 3.9–500 mg/L. Red wine samples were diluted twofold (1:1), whereas the white wine samples did not require dilution based on preliminary experiments.

#### 2.3.3 Antioxidant activity

DPPH free radical scavenging assay was followed for the determination of antioxidant capacity (AOC) of wine samples (Moreno‐Arribas & Polo, 2009). In brief, 800 μl of DPPH solution in methanol (0.004% w/v) was added to a 50 μl of wine sample previously diluted wine samples (10‐fold dilution for red wines and fivefold dilution for white wines). A control experiment consisted of 50 μl of methanol and 800 μl of DPPH. The mixtures were then incubated for 30 min at room temperature in dark. Absorbance of sample (*A*_s_) was measured at 515 nm against a methanol blank (*A*_c_). Percentage inhibition was calculated using the formula.

$$\%\;\text{Inhibition}=\frac{A_{c}-A_{s}}{A_{c}}\times 100$$

The AOC was expressed in mg/L ascorbic acid equivalent (AAE) using a calibration curve in the concentration range 10–120 mg/L.

#### 2.3.4 Total tannins content

The total tannins content (TTC) was determined by following a protocol described previously (Hosu et al., 2014) with volume adjustment. Two samples were prepared for each wine containing 200 μl of 10‐fold‐diluted wines, 300 μl of concentrated HCl, and 100 μl of distilled water. The first sample was incubated at 100°C for 30 min, whereas the second sample was left at room temperature with the addition of 50 μl ethanol. Absorbance of both samples was measured at 470, 520, and 570 nm. The differences in absorbance between two samples at a given wavelength were calculated and are represented as Δ*A*_470_, Δ*A*_520_, and Δ*A*_570_. The Δ*A*_470_ and Δ*A*_570_ were then expressed in terms of Δ*A*_520_ using following formula: Δ*A*_520_ = 1.1 × Δ*A*_470_ and Δ*A*_520_ = 1.54 × Δ*A*_470_. The lowest Δ*A*_520_ value was chosen to estimate total tannin content as g/L of wine using following equation (Hosu et al., 2014): TTC = 15.7 × lowest Δ*A*_520_.

#### 2.3.5 Total anthocyanin content

The total anthocyanin content (TAC) was quantified using a SO_2_ bleaching protocol (Moreno‐Arribas & Polo, 2009). Two mixtures were prepared for each wine sample containing 50 μl wine, 50 μl HCl in ethanol (0.1%), and 100 μl aqueous HCl (20%). Then, 220 μl of distilled water was added to the first mixture, whereas the same amount of sodium bisulfite (26%) was added to the second one. The mixtures were then diluted (1:1). Absorbance of both mixtures was measured at 520 nm against a blank (50 μl HCl in ethanol (0.1%), 100 μl aqueous HCl (20%), and 270 μl distilled water). Differences in the absorbance between two mixtures were calculated as Δ*A*_520_. The TAC as mg/L of wine was quantified using following equation: TAC = 875 × Δ*A*_520_.

#### 2.3.6 Carbohydrate content

The total carbohydrates content (TCC) in wine samples was measured by anthrone‐sulfuric acid assay method (Dreywood, 1946). A stock solution of glucose (20 g/L) was prepared in distilled water. The stock solution was serially diluted to prepare a calibration curve. A 0.2% anthrone was prepared in concentrated sulfuric acid. Sample and standard solution (200 μl) were mixed with anthrone‐sulfuric acid reagent (400 μl) in an Eppendorf tube. The mixture was then vortexed, and an aliquot of the mixture was transferred into 96‐well plates. Absorbance readings were recorded at 625 nm. The carbohydrate in wine sample was estimated using a linear regression equation obtained from a calibration curve ranging from 0.039 to 0.625 mg/ml.

#### 2.3.7 Color parameters

We estimated color parameters by measuring absorbance of wine samples at wavelengths 420, 520 and 620 nm (Gómez‐Plaza, Gil‐Muñoz, López‐Roca, & Martínez, 2000; Kalkan Yildirim, 2006; Ortiz, Marín‐Arroyo, Noriega‐Domínguez, Navarro, & Arozarena, 2013). Sum of absorbance at 420 and 520 nm was expressed as color density (IC), whereas sum of absorbance at 420 nm, 520 nm, and 620 nm was referred to as color intensities (IC’). The proportions of red (%R), yellow (%Y), and blue (%B) were determined by using following formula (Kalkan Yildirim, 2006).

$$\%\;R=[\left(A_{520}\times 100\right)/{\text{IC}}^{\prime }]$$

$$\%\;Y=[\left(A_{420}\times 100\right)/{\text{IC}}^{\prime }]$$

$$\%\;B=[\left(A_{620}\times 100\right)/{\text{IC}}^{\prime }]$$

Wine color (WC) was determined by adding 20 μl acetaldehyde to 2 ml wine and measuring its absorbance at 520 nm after 45 min. Wine total color of pigments (WCP) was determined by adding 900 μl HCl (0.1N) to 100 μl wine and measuring its absorbance value at 520 nm after 4–5 hr. Wine polymeric pigment color (WPPC) was determined by adding 15 mg sodium bisulfite to 5 ml wine and measuring its absorbance at 520 nm after 1 min (Kalkan Yildirim, 2006).

### 2.4 Statistical analysis

Each experiment was repeated three times, and the results are reported as mean ± standard deviation. Pearson product‐moment correlation was used for the analysis of correlation between various parameters and significance of correlation. We also performed principal component analysis (PCA) to better understand the correlation. All analyses and figures were done in R version 3.4.2.

## 3 RESULTS AND DISCUSSION

### 3.1 Total phenol, total flavonoids, and antioxidant capacity

Phenolic compounds are responsible for color and astringency of wines. Polyphenolic components of wine are broadly categorized into flavonoids and nonflavonoids as well as polymeric pigments such as anthocyanins. Flavonoids are the predominant class of compounds accounting for approximately two‐thirds of the dietary phenols (Moreno‐Arribas & Polo, 2009). The flavonoid content of red wine has been suggested as an explanation for the low incidence of coronary heart disease, despite having a diet high in fat and being heavy smokers in French people. Therefore, determination of phenolics in wines is on rise both in food science and in medicine (Cordova & Sumpio, 2009).

**Figure 1:**
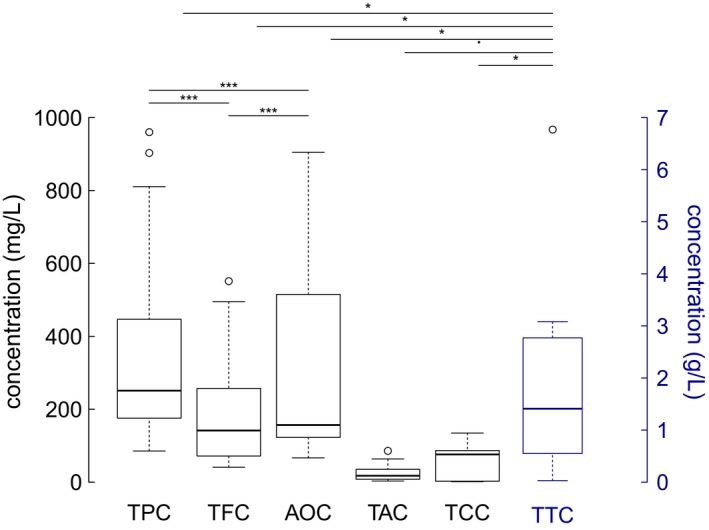
Box plot of various wine parameters. TPC: total phenolic content, TFC: total flavonoid content; AOC: total antioxidant activity; TAC: total anthocyanin content; TTC: total tannin content; TCC: total carbohydrate content. Bars on top of box plots show significant pairwise comparisons. Stars indicate level of significance (^†^ *p* < 0.1, *p < 0.05, **p < 0.01, ***p < 0.001). See Table 3 for statistical details

The TPC is an important parameter widely used for evaluation of wines and other foods. We measured the TPC of Nepali wine samples using the well‐known Folin–Ciocalteu assay. Similarly, we evaluated the TFC using aluminum chloride method. The TPC, TFC, and AOC varied widely with wine samples (Table 2). TPC ranged from 85.5 ± 2.9 to 960 ± 48.7 (mean = 360.5 ± 268.7) mg/L GAE (Figure 1). Highest amount of TPC was found in W16R, followed by W13R and W17R, whereas the lowest was found in W2W. TFC ranged from 40.9 ± 2.2 to 551.3 ± 41.1 (mean = 188.9 ± 161.5) mg/L QE (Figure 1). The highest TFC was found in W16R, followed by W17R, W3R, and W13R, whereas the lowest was found in W2W (*see* Table 1 for sample description). Red wine samples had significantly higher average TPC (463 ± 290 vs. 183.8 ± 66.4 mg/L, *p *<* *0.05) and TFC (264.2 ± 160.4 vs. 59.8 ± 16.8 mg/L, *p *<* *0.01) than the white wine samples. The concentration and composition of the phenolics present in wines depend largely on the source of fruit, environmental factors in vineyard, and the method of wine‐making (Cheynier et al., 2006; Frankel, Waterhouse, & Teissedre, 1995). Most of the phenolic compounds are concentrated in the grape skin, and therefore, greater concentrations of phenolics can be expected in red wines. Our results confirm a variation in phenolic content among wine samples tested. These results are in agreement with the available literature (Frankel et al., 1995; Simonetti, Pietta, & Testolin, 1997).

**Table 2:** Total phenolic content (TPC), total flavonoids content (TFC), total antioxidant activity (AOC), total anthocyanins content (TAC), total tannins content (TTC), total carbohydrate content (TCC) and color parameters of wine samples

Sample ID	TPC (mg/L)	TFC (mg/L)	TAA (mg/L)	TAC (mg/L)	TTC (g/L)	TCC (g/L)	WC (A.U.)	WPPC (A. U.)	WCP (A. U.)	Red (%)	Yellow (%)	Blue (%)
W01R	133.1 ± 14.7	86.3 ± 3.9	131.7 ± 12.5	3.2 ± 1.8	0.03 ± 0.0	1.97 ± 0.09	0.454 ± 0.008	0.488 ± 0.003	0.085 ± 0.004	45.5 ± 0.1	48.2 ± 0.1	6.4 ± 0.2
W02W	85.5 ± 3.0	40.9 ± 2.2	66.6 ± 9.8	ND	ND	1.97 ± 0.04	0.076 ± 0.003	0.115 ± 0.006	0.052 ± 0.002	27.3 ± 0.3	51.1 ± 0.5	21.6 ± 0.2
W03R	531.2 ± 3.2	391.4 ± 13.7	667.2 ± 4.6	22.8 ± 3.5	0.48 ± 0.1	3.37 ± 0.12	0.579 ± 0.011	0.507 ± 0.005	0.128 ± 0.006	44.9 ± 0.8	41.4 ± 0.2	13.7 ± 1.0
W04W	284.0 ± 16.8	82.7 ± 8.0	343.4 ± 8.6	ND	ND	2.06 ± 0.05	0.073 ± 0.004	0.072 ± 0.003	0.053 ± 0.003	29.3 ± 1.1	45.9 ± 1.7	24.9 ± 0.7
W05R	297.6 ± 3.3	201.9 ± 14.0	281.1 ± 12.5	30.3 ± 4.0	2.62 ± 0.1	97.49 ± 1.15	0.661 ± 0.005	0.532 ± 0.014	0.161 ± 0.001	50.2 ± 0.1	37.6 ± 0.1	12.2 ± 0.1
W06W	173.1 ± 11.1	49.6 ± 6.7	96.2 ± 13.1	ND	1.41 ± 0.2	76.52 ± 0.88	0.060 ± 0.005	0.058 ± 0.002	0.055 ± 0.002	30.0 ± 0.2	43.9 ± 0.6	26.1 ± 0.7
W07R	203.8 ± 11.3	141.8 ± 7.1	134.8 ± 12.7	10.8 ± 4.8	0.35 ± 0.1	1.31 ± 0.07	0.453 ± 0.007	0.461 ± 0.008	0.107 ± 0.002	37.4 ± 0.1	46.5 ± 0.0	16.1 ± 0.1
W08W	148.8 ± 3.5	82.9 ± 3.3	87.5 ± 14.4	ND	0.06 ± 0.1	1.38 ± 0.12	0.120 ± 0.001	0.106 ± 0.002	0.059 ± 0.003	28.0 ± 0.2	54.9 ± 0.6	17.1 ± 0.4
W09R	251.0 ± 5.8	93.4 ± 13.1	156.8 ± 19.9	5.0 ± 3.1	3.01 ± 0.1	109.11 ± 2.71	0.411 ± 0.021	0.358 ± 0.008	0.106 ± 0.015	42.1 ± 0.2	46.2 ± 0.1	11.6 ± 0.1
W10R	362.6 ± 18.7	144.9 ± 8.3	263.7 ± 45.5	85.8 ± 3.8	2.77 ± 0.2	79.54 ± 1.04	0.597 ± 0.006	0.314 ± 0.003	0.227 ± 0.014	45.8 ± 0.2	42.2 ± 0.2	12.0 ± 0.0
W11W	197.4 ± 6.1	60.9 ± 4.2	140.7 ± 11.5	ND	0.81 ± 0.1	86.15 ± 4.79	0.112 ± 0.006	0.115 ± 0.001	0.065 ± 0.008	28.0 ± 0.3	55.6 ± 0.9	16.3 ± 0.5
W12R	360.5 ± 13.0	202.5 ± 6.8	362.7 ± 17.3	40.3 ± 4.5	1.36 ± 0.1	24.51 ± 0.68	0.408 ± 0.005	0.292 ± 0.002	0.131 ± 0.009	44.3 ± 0.1	43.8 ± 0.2	11.9 ± 0.2
W13R	902.9 ± 38.7	399.4 ± 17.6	905.0 ± 6.8	25.7 ± 11.6	2.98 ± 0.1	92.27 ± 2.94	1.827 ± 0.007	1.823 ± 0.034	0.235 ± 0.009	39.2 ± 0.0	52.7 ± 0.1	8.1 ± 0.1
W14R	178.1 ± 37.9	150.7 ± 11.7	130.7 ± 11.7	8.5 ± 3.1	0.55 ± 0.0	87.32 ± 0.55	0.684 ± 0.004	0.728 ± 0.006	0.111 ± 0.001	31.6 ± 0.1	58.9 ± 0.4	9.5 ± 0.3
W15R	571.0 ± 12.0	312.0 ± 12.9	679.1 ± 4.6	12.5 ± 7.1	3.08 ± 0.1	76.20 ± 1.22	1.307 ± 0.018	1.301 ± 0.003	0.182 ± 0.007	37.7 ± 0.1	52.9 ± 0.2	9.4 ± 0.2
W16R	960.0 ± 48.7	551.3 ± 41.1	847.9 ± 4.6	7.6 ± 6.1	1.81 ± 0.0	3.47 ± 0.12	0.935 ± 0.017	0.952 ± 0.012	0.150 ± 0.013	39.4 ± 0.0	48.6 ± 0.1	2.0 ± 0.1
W17R	810.5 ± 33.3	494.9 ± 18.1	841.1 ± 13.7	63.6 ± 6.6	6.77 ± 0.3	85.32 ± 4.10	1.916 ± 0.036	1.376 ± 0.006	0.320 ± 0.011	47.1 ± 0.2	41.3 ± 0.0	11.6 ± 0.2
W18W	247.9 ± 4.9	48.6 ± 3.7	114.8 ± 8.0	ND	1.60 ± 0.4	134.74 ± 1.83	0.064 ± 0.003	0.076 ± 0.004	0.063 ± 0.005	27.4 ± 1.3	50.0 ± 3.5	22.6 ± 2.2
W19W	150.2 ± 7.2	53.0 ± 8.6	72.6 ± 5.2	ND	0.69 ± 0.3	38.74 ± 2.29	0.060 ± 0.001	0.089 ± 0.005	0.051 ± 0.005	28.3 ± 0.5	47.4 ± 1.3	24.4 ± 0.9

Values expressed as mean ± SD; SD = standard deviation (*n* = 3); Wine C = wine color; Wine PPC = Wine polymeric pigment content; Wine CP = wine color pigment; ND = not detected.

The phenolic compounds are also considered a major contributor to the sensory characteristics and antioxidant activity of wines. Therefore, we also evaluated the AOC of wine samples using one of the widely used methods for detecting antioxidant activity, the DPPH scavenging method. In this assay, antioxidants present in wine samples react with DPPH radical and produce yellow α,α‐diphenyl‐β‐picrylhydrazine molecule. The degree of discoloration indicates the radical scavenging activity of the antioxidant (Amorati & Valgimigli, 2015). The phenols and polyphenolic compounds in wine samples trap two or more peroxyl radicals based on the size of the antioxidant molecule while undergoing this decolorization reaction (Boskou, 2010). The AOC of wine samples ranged from 66.6 ± 9.8 to 904.9 ± 6.8 (mean = 332.8 ± 296.5) mg/L AAE (Figure 1). Wine sample W13R had the highest antioxidant property followed by W16R and W17R, whereas wine sample W2W had the lowest AOC (*see* Table 2). Similar to TPC and TFC, the average AOC in red wines (450.1 ± 312.9 mg/L) was significantly higher (*p *<* *0.05) than in the white wines (131.7 ± 96.7 mg/L) as found in other studies (Khanam, Oba, Yanase, & Murakami, 2012). Our results confirm that the higher the concentration of antioxidant, the higher is the free radical scavenging activity. Interestingly, wines that are made up of grapes were found to have relatively higher antioxidant potential (e.g., W13R, W16R, and W17R, although not statistically significant), whereas wines made from other fruits and berries were found to contain lesser antioxidant potential similar to study reported in other studies (Frankel et al., 1995; Lehtonen, Rokka, Hopia, & Heinonen, 1999). Similarly, TPC and TFC were found to be relatively higher on wines made from grapes than those made from other fruits and berries. Measured values of each parameter are given in Table 2.

We also examined the correlation of phenolic content and AOC of the samples. TPC and TFC showed strong positive correlation (*r* = 0.946, *p* < 0.001). Average TFC was 59.3% of TPC in case of red wines and 35.3% in case of white wines. Red wines are normally made from whole fruit unlike white wines, which are made from fruit pulps only (Mills, Phister, Neeley, & Johannsen, 2008). High amount of phenolic and flavonoid compounds such as flavonols, anthocyanidins, proanthocyanidins, catechins, tannins, and stilbenoids is abundantly present in skin and seeds of fruits, which may account for higher presence of phenols and flavonoids, with higher percent amount of flavonoids in total phenols in red wines compared to white wines (de Camargo, Regitano‐d'Arce, Biasoto, & Shahidi, 2014). Wines with higher TPC are considered to be better quality (Mercurio, Dambergs, Cozzolino, Herderich, & Smith, 2010). It was noted that the highest amount of TPC and TFC was found in W16R, which was made from grapes with added natural palm and pears. In contrary, the least amount was found in W2W, which was made from the mixture of fruits, roots, herbs, and honey. The antioxidant property of wine samples strongly correlated with phenolic content (*r* = 0.971, *p* < 0.001) and flavonoid content (*r* = 0.949, *p* < 0.001) (*see* Table 3). Our results suggest that phenolic compounds are a major contributor to antioxidant capacity in wines. Similar results have also reported a linear relationship between antioxidant capacity and total phenols (Deighton, Brennan, Finn, & Davies, 2000).

**Table 3:** (a) Pearson correlation coefficient values. (b) *p*‐values

(a)							
	TPC	TFC	AOC	TAC	TTC	TCC	WC
TPC	1						
TFC	0.946	1					
AOC	0.971	0.949	1				
TAC	0.181	0.121	0.160	1			
TTC	0.605	0.533	0.585	0.544	1		
TCC	0.090	−0.042	0.013	0.283	0.515	1	
WC	0.835	0.827	0.847	0.248	0.740	0.256	1

(b)						
	TPC	TFC	AOC	TAC	TTC	TCC
TPC						
TFC	8.7.20E‐10					
AOC	4.62E‐12	5.72E‐10				
TAC	0.574	0.706	0.619			
TTC	0.010	0.027	0.0137	0.067		
TCC	0.713	0.864	0.957	0.373	0.034	
WC	8.69E‐06	1.24E‐05	4.63E‐06	0.436	6.75E‐4	0.289

### 3.2 Total anthocyanin and tannin content

Anthocyanins present in wines are water‐soluble flavonoid pigments that are present in almost all tissues of higher plants including roots, stems, leaves, flowers, and fruits. In grapes, they are located mainly in the skin and in the flesh. Monomeric anthocyanins in young red wines contribute the majority of color of the wine (He et al., 2012a). During wine maturation and aging, these monomeric anthocyanin compounds form complex and stable anthocyanin‐derived pigments resulting in a variation of color (He et al., 2012b). Similarly, tannins are a group of bulky phenolic compounds that attribute to red wine mouthfeel and astringency perception by interacting with salivary proteins in mouth. The wine astringency decreases over the aging process. Wine tannin content and composition vary on the type of grape and wine‐making conditions (Cheynier et al., 2006).

We measured total anthocyanin content (TAC) and total tannin content (TTC) in Nepali wines. The TAC in red wines ranged from 3.2 ± 1.8 to 85.7 ± 3.8 mg/L (mean = 26.3 ± 25.6 mg/L, Figure 1, Table 2), whereas the TTC ranged from 0.03 ± 0.01 to 6.8 ± 0.28 (mean = 1.79 ± 1.68) g/L. Our findings also show that wine made from grapefruit shows relatively high TAC. The highest TAC observed in W17R is made from tempranillo grape, whereas W4W made from aiselu (Himalayan Raspberry) did not contain detectable amount of anthocyanins. Wines made from apple, lychee fruits, roots, herbs, and honey also showed poor TAC. The tannin was not detected in samples W2W and W4W. Highest TTC was found in W17R followed by W9R and W15R. Most white wine samples were found to contain lower levels of tannins (nd to 1.6 g/L), and red wine samples were found to contain significant amount of tannins (0.35–6.8 g/L). This observation is in accordance with our observation of high TPC and TFC in red wines compared to white wines as reported earlier. High tannin content observed in red wines can be correlated to the fact that seeds contribute hugely to the tannin contents and red wines are made from whole fruits, including seeds (Vernon L. Singleton & Trousdale, 1992). To the contrary, white wines are made from fruit pulp only, which may account for its poor TAC. Polymeric tannins, which are responsible for wine astringency, bitterness, and complexity, are reported to be found in high amount in seeds of grapefruit especially (V. L. Singleton & Draper, 1964). Amount of tannins may also impact in‐mouth sensory properties of wine (McRae, Schulkin, Kassara, Holt, & Smith, 2013), and higher amount of tannin indicates better grade wine (Kassara & Kennedy, 2011; Mercurio et al., 2010). The TTC may be useful to grade the wines in terms of their quality (McRae et al., 2013). In addition, higher amount of TAC is also found to be correlated to higher market value (Mercurio et al., 2010).

TAC was poorly correlated with TTC (*r* = 0.544, *p* = 0.067), TPC (*r* = 181, *p* = 547), TFC (*r* = 0.121, *p* = 0.706), and AOC (*r* = 0.160, *p* = 0.619). The tannin content was correlated with TPC (*r* = 0.605, *p* = 0.010), TFC (*r* = 0.533, *p* < 0.05), and AOC (*r* = 0.585, *p* < 0.05) (*see* Table 3). These correlations are similar to literature reports.

### 3.3 Carbohydrates

Carbohydrates are minor components of wine. They are responsible for sensorial properties and play role during fermentation. Some low molecular sugars such as reducing sugars—glucose and fructose—are common in red wines. These may remain in wine after fermentation. Monosaccharides such as galactose, arabinose, ribose, rhamnose, and xylose are also present in wine. In addition, red wines contain disaccharides such as sucrose and trehalose. Polysaccharides present in wine are derived both from cell walls of micro‐organisms or grapes (Moreno‐Arribas & Polo, 2009). We measured total carbohydrates present in the wine samples using Anthrone method. The total carbohydrate content (TCC) in wine samples ranged from 1.31 ± 0.07 g/L to 134.78 ± 1.83 g/L (mean = 52.81 ± 45.66) (*see* Figure 1 and Table 2). These results are comparable to other studies as reported: for example, 49 g/L in orange wine (Kelebek, Selli, Canbas, & Cabaroglu, 2009) to 10–130 g/L (Kupina & Roman, 2014) but lower than as reported by Alamo et al. (200 g/L) (del Alamo, Bernal, & Gómez‐Cordovés, 2000). The mean carbohydrate content in red wines was not statistically different (*p* = 791) from the mean carbohydrate content in white wine samples tested. The carbohydrate content was not correlated with other parameters measured except TTC (*r* = 0.515, *p* < 0.05) (*see* Table 3).

### 3.4 Color parameters

Color parameters of red wines were analyzed in terms of wine color (WC), wine total color of pigments (WCP), and wine polymeric pigment color (WPPC). The WC parameter measures overall wine color. The WCP parameter measures color due to ionized anthocyanins, which are mainly responsible for the color of young wines, whereas the WPPC parameter measures color of polymeric pigments. Sodium bisulfite used in the assay of WPPC bleaches most of the color of wine except the color of polymeric pigments formed by condensation reaction between anthocyanins and other phenolic compounds. WPPC may tell us about the age of wines as polymeric pigments measured in this assay are formed as the wine age (Ortiz et al., 2013).

Measured values of WC, WCP, and WPPC parameters in various red wine samples are listed in Table 2. WC ranged from 0.060 ± 0.005 to 1.916 ± 0.036 (mean = 0.57 ± 0.57) (A.U.) with high values in W17R and W13R followed by W15R. The values for WCP ranged from 0.051 ± 0.005 to 0.320 ± 0.011 (mean = 0.123 ± 0.075) (A.U.). The WCP was found to be high in W17R followed by W15R and W10R. Similarly, the WPPC ranged from 0.058 ± 0.002 to 1.823 ± 0.034 (mean = 0.541 ± 0.511) (A.U.) and was found to be high in W13R followed by W17R and W15R. We also measured the wine color in terms of percentage red, yellow, and blue (Table 2). We found that wine color was mostly dominated by red and yellow color with little percent of blue color. Also, wine with high percentage yellow color has low percentage red color and vice versa. The proportion of red color, which mainly arises from anthocyanins, decreases as anthocyanin polymerizes with tannins and other phenolic compounds leading to the increase in the proportion of yellow color (Pascal Ribéreau‐Gayon, Dubourdieu, Donèche, & Lonvaud, 2006; Sarni, Fulcrand, Souillol, Souquet, & Cheynier, 1995). This process increases as the wine age.

The WC showed strong positive correlation with TPC (*r* = 0.835, *p* < 0.001), TFC (*r* = 0.827, *p* < 0.001), AOC (*r* = 0.847, *p* < 0.001), and TTC (*r* = 0.740, *p* < 0.001), however, showed insignificant correlation with TAC (*r* = 0.248, *p* = 0.436) and TCC (*r* = 0.256, *p* = 0.289). The nonsignificant correlation of WC with TAC indicates that the wine color was contributed by other factors besides anthocyanin content. In addition to TAC, wine color depends upon compounds that bound with anthocyanins and polymeric pigments. Concentration of such compounds varies with different factors including age, varietal location, climatic condition, soil pH, nutrient availability, temperature, and season (Boulton, 2001; Stratil et al., 2008). Therefore, wine color is also considered as a product of the collective contribution of many different variables. Even though tannins are usually responsible for wine taste, bitterness, astringency, and complexity, they can also indirectly contribute for wine color by polymerizing with anthocyanin and forming polymeric pigments (Vernon L. Singleton & Trousdale, 1992; Vidal et al., 2004). Kassara et al. reported an increase in wine color with total tannin concentration (Kassara & Kennedy, 2011).

It is interesting to note, as shown by correlation tests, that wine samples with high WC have relatively high phenolic and flavonoid content.

We also evaluated the relationship of wine price with various wine quality parameters. The wine price was not correlated with phenol (*r* = −0.088, *p* > 0.05), flavonoid (*r* = −0.088, *p* > 0.05), antioxidant capacity (*r* = −0.088, *p* > 0.05), and color (*r* = 0.159, *p* > 0.5). However, wine prices showed moderate correlation with tannins content (*r* = 0.457, *p* < 0.05) and anthocyanin content (*r* = 0.719, *p* < 0.01). However, previous studies have shown that the wine price correlated with wine grade reflected by the amount of phenolic compounds (e.g., tannin; Kassara & Kennedy, 2011).

### 3.5 Principal component analysis

**Figure 2:**
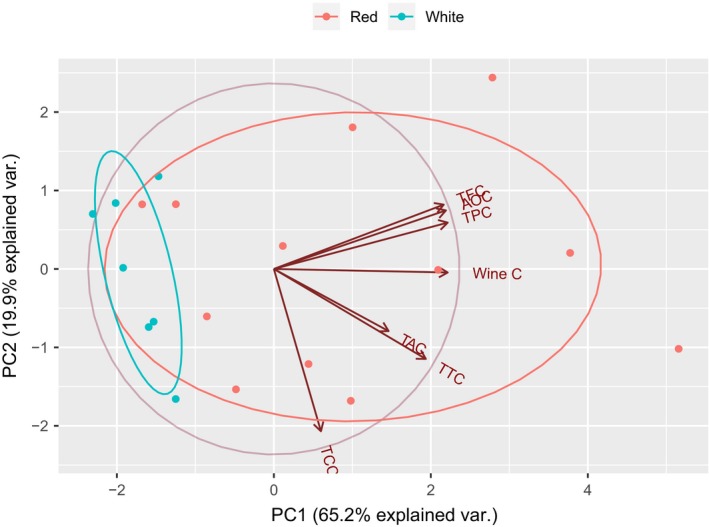
Principal component analysis plot of various parameters measured in wine samples

Principal component analysis (PCA) was performed on wine samples using five variable components: phenols, flavonoids, antioxidants, tannins, and anthocyanins to analyze the structure of correlation matrix. KMO & Bartlett's test (KMO measure: 0.795, approx. chi‐square: 98.82, *p* < 0.001) signifies that the variables are significantly correlated, and the dataset is valid for PCA. It was found that PC1 (eigenvalue = 3.675) accounts for 65.2% of total variance in dataset and PC2 (eigenvalue = 0.911) accounts for 19.9% of total variance. From component loading matrix, it was observed that phenols (*ρ* *=* 0.953), flavonoids (*ρ* = 0.938*)*, and antioxidants (*ρ* = 0.944) load maximum to PC1, signifying that PC1 best explains the variability of phenol, along with flavonoids and antioxidants variables among the wine samples. Likewise, anthocyanin (*ρ* = 0.708) loads maximum to PC2, signifying that PC2 best explains the variability of anthocyanins among different wine samples. Both PC1 and PC2 contributed to explain the variability of tannins in wine samples (*ρ* = 0.781 with PC1, *ρ* = 0.429 with PC2). Figure 2 shows the PCA plot of wine samples showing scattering of sample according to different PC scores and projection of original variables on to the principal components. Wine samples are observed to scatter around the biplot as a function of different variables’ content. The wine samples toward the projection of variables show high content of those variables. Accordingly, W16R, W15R, W13R, and W3R, which lie toward the projection of phenols, flavonoids, and antioxidants, show high content of these variables. Likewise, W10R, which lies toward the projection of anthocyanin, shows highest content of anthocyanin, and W17R, which lies toward the projection of tannins, shows highest tannins’ content. Also, it was observed that the projection of phenol, flavonoids, and antioxidants is closely aligned signifying a strong correlation between these parameters. The distribution of red and white wine samples along the two principal components shows good discrimination of the wine samples. It can be observed that red wines and white wines are clearly separated by first principal component, with majority of red wines but not white wines lying toward the direction of projection of variables, suggesting that red wines have higher variable contents: phenol, flavonoids, antioxidants, anthocyanins, and tannins than white wines.

### 3.6 Comparison of Nepali wines with other wines in world market

Phenolic compounds are the major secondary metabolites found in wines including flavonoids, anthocyanins, and tannins, which also correlate significantly with antioxidant activity. These secondary metabolites consist of large number of compounds with high variation in their medicinal values (Rice‐Evans et al., 1997). The phenolic analyses are frequently used effective tools in characterizing different wines. Generally, TPC and TTC are positively correlated with wine quality (Mercurio et al., 2010). Therefore, we have compared Nepali wines with wines manufactured in other countries as reported in the literature in terms of TPC, TAC, and TTC (Table 4). The phenolic content in red wines in our study was about three to 10 times lower than the red wines from other countries. However, the phenolic content in white wines in our study was found to be comparable to the white wines from other countries. The variation in phenolic content of wines from various countries is obvious as the phenolic content of wine depends upon several factors such as raw materials used, their species and variety, climate, vinification procedure, aging, and storage (Stratil et al., 2008). Similarly, the total anthocyanin content in our study was found to be 5–29 times lower than in samples from other countries (*see* Table 4).

**Table 4:** Comparison of Nepali wines with wines produced in other countries

Country	Wine type	TPC (mg/L GAE)	TAC (mg/L)	TTC (g/L)
Nepal (this study)	Red	463.5 ± 290.4 (*n* = 12)	26.3 ± 25.7 (*n* = 12)	2.1 ± 1.9 (*n* = 12)
	White	183.8 ± 66.4 (*n* = 7)	–	0.6 ± 0.7 (*n* = 7)
Australia (Bindon et al., 2014; Yoo et al., 2011)	Red	2517.4 ± 467.6 (*n* = 55)	786.1 ± 240.8 (*n* = 39)	1.2 ± 0.6 (*n* = 39)
	White	–	–	–
Brazil (Gris et al., 2013; Ishimoto, Ferrari, Bastos, & Torres, 2006; Lucena et al., 2010)	Red	5087.5 ± 865.9 (*n* = 8)	47.4 ± 42.2 (*n* = 8)	–
	White	305.0	–	–
China (Jiang & Zhang, 2012; Li et al., 2009)	Red	2068.5 ± 441.6(*n* = 24)	119.7 ± 59.3 (*n* = 24)	1.7 ± 0.4 (*n* = 8)
	White	301.8 ± 85.9 (*n* = 11)		
Czech Republic (Stratil et al., 2008)	Red	1544.8 ± 358.8 (*n* = 21)	–	–
	White	115.5 ± 22.9 (*n* = 8)	–	–
Italy (Cassino et al., 2016; Garaguso & Nardini, 2015; Simonetti et al., 1997)	Red	4321.1 ± 873.5 (*n* = 16)	204.8 ± 172.8 (*n* = 36)	–
	White	119.3 ± 25.8 (*n* = 3)	–	–
Portugal (Cristino, Costa, Cosme, & Jordão, 2013; Paixão, Perestrelo, Marques, & Câmara, 2007)	Red	1842.2 ± 77.4 (*n* = 5)	238.8 ± 84.6 (*n* = 20)	1.7 ± 0.3 (*n* = 20)
	White	369.4 ± 55.2 (*n* = 5)	–	–
Romania (Hosu et al., 2014, 2016)	Red	2455.9 ± 761.5(*n* = 27)	140.2 ± 59.6(*n* = 27)	1.4 ± 0.5(*n* = 27)
	White	255.6 ± 64.7 (*n* = 27)		
Spain (Gómez‐Plaza et al., 2016; Sánchez‐Moreno, Larrauri, & Saura‐Calixto, 1999)	Red	1613.2 ± 460.2 (*n* = 8)	329.9 ± 96.9 (*n* = 14)	2.3 ± 0.9 (*n* = 14)
	White	240.8 ± 42.7 (*n* = 5)	–	–

## 4 CONCLUSIONS

In this study, we measured major chemical and color parameters of wines manufactured in Nepal. Similar to other studies in the literature, the secondary metabolites were found to be significantly higher in red wines compared to white wines, and the phenolic and flavonoid compounds were correlated to each other and to wine color. The secondary metabolites, specially the phenolic compounds, are the major contributors of medicinal values of wines. Even though Nepali wines are perceived as high‐valued wines considering raw materials from high altitude, we found that the medicinally important secondary metabolites (e.g., phenolic) are three to 10 times lower compared to the red wines produced in many other countries. Interestingly, the total anthocyanin content, which is considered to be responsible for the color of wine, in our study, was found to be 5–29 times lower than in samples from other countries and did not correlate with wine color. Unlike many international wines, the price of Nepali wine did not correlate with the phenolic content. Some of the wine samples did not clearly mention the raw material on the bottle. In addition, even though the wine was produced in relatively high elevation places, it was not clear whether the raw materials were produced locally or purchased from other areas. This may require further investigation with molecular characterization. Our research may help Nepali wine producers to consider ways to upgrade the quality of wines and government agencies to come up with necessary policies.
